# Tolerogenic citrullinated peptide for arthritis

**DOI:** 10.18632/oncotarget.5016

**Published:** 2015-07-26

**Authors:** Smadar Gertel, Yehuda Shoenfeld, Howard Amital

**Affiliations:** Department of Medicine, Sheba Medical Center, (Affiliated to Tel-Aviv University), Tel-Hashomer, Israel

**Keywords:** rheumatoid arthritis, tolerance

Disease-specific autoantibodies directed against post-translation citrullinated proteins/peptides (ACPAs) are specific serological markers for rheumatoid arthritis (RA) and have been accepted as one of the classification criteria for the disease. Beyond diagnosis these autoantibodies have also been shown also to predict disease severity. These autoantibodies can be detected several years prior to the clinical onset of RA, with increasing titers overtime, as disease onset is established.

Citrullination is a post-translational modification of proteins in which arginine is converted into citrulline, an unnatural amino acid which is not encoded by DNA. The reaction is catalyzed by calcium-dependent peptidyl arginine deiminase (PAD). Citrullinated proteins have been detected in the synovial membranes and synovial fluid of patients with various forms of arthritis. Protein citrullination has been shown to be involved in immune tolerance breakdown in experimental autoimmune arthritis. Only small amounts of citrullinated proteins is found in the normal synovial tissue, whereas active citrullination has been clearly associated with RA. In addition, ACPAs are strongly associated with increased risk of developing RA in healthy individuals. Their presence in RA patients is associated with a more erosive arthritis and poorer response to therapy [1].

It is reasonable to assume that citrullinated peptides are among the earliest arthritic autoantigens that may be suitable as potential candidates for specific immune tolerance induction. The finding of an immunodominant citrullinated peptide as antigen-specific immunomodulatory agents capable for restoration of the altered autoimmune response in RA seems to be an interesting target for intercepting the disease.

The possible mechanisms by which autoantigens or their derivatives induce specific tolerance are based on frequent administrations of low dose soluble antigens. By these means administration of a synthetic peptide could manipulate the interaction of antigen presenting cells (APCs) with lymphocytes. This tolerogenic presentation displayed on MHC class II-expressing T cells (T-APC) induces partial signals in Ag-specific T cell clones including deletion, anergy and suppression of the autoreactive cells.

Administration of a citrullinated filaggrin peptide as a prophylactic measure to prevent collagen induced arthritis prior to induction in DBA mice has been shown to be successful and to protect mice from developing arthritis [2]. This study reasoned this finding by the ability of the citrullinated peptide to bind ACPA and to induce tolerance of citrullinated-peptide specific T cells.

However, one of the difficulties in developing effective tolerogenic peptides for immune tolerance strategies in RA is the selection of a specific antigen since the autoimmune response is against multiple targets; diverse citrullinated proteins in different synovial structures. The question is, therefore, which specific citrullinated autoantigens drives the immune response in RA and whether those antigens are the best to be employed for immune tolerance restoration. It was shown that the most prevalent citrullinated autoantigens, in arthritic joints are: citrullinated fibrinogen, vimentin, collagen type II and α-enolase [3].

Therefore we constructed a multi-antigenic peptide for immune tolerance specific for RA. The peptide is composed of sequence of known citrullinated autoantigens (filaggrin, β-fibrinogen, collagen type II and vimentin) in analogy to the mix of citrullinated peptides that are used currently in diagnostic sera tests. One of our peptide comparator was a short filaggrin peptide composed of five amino acid [4]. This minimal filaggrin peptide with citrulline residue in it, still specifically recognized by RA sera. The ability of the short peptide to recognize specific ACPA indicated its ability to modulate citrulline specific autoreactive T cells as well.

The multi epitope citrullinated peptide we tailored termed Cit-ME was injected to adjuvant induced arthritis rats and was shown to attenuate arthritis manifestations [5]. The amelioration in clinical signs of arthritis was related to up regulation of T regulatory cell subset and an elevated apoptosis rate of T-cells associated with reduced Th17 population.

**Figure 1:**
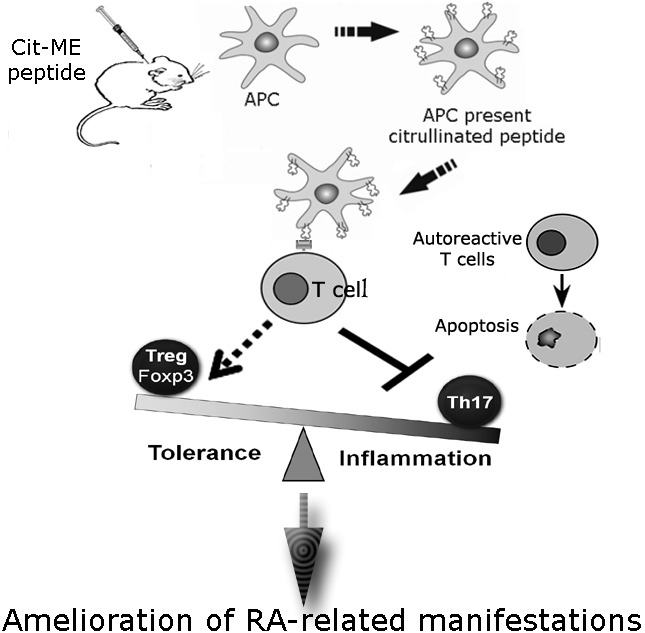
Putative mechanisms of action of the Cit-ME following administration to adjuvant induced arthritis rats are shown

The putative mechanisms of the Cit-ME peptide are demonstrated in Figure 1.

Another option that remains to be explored is the possibility to employ tolerance induction by oral administration of the peptide. It was shown that oral administration of peptide derived from collagen type II (250-270) was able to suppress the cellular and humoral immune response in collagen-induced arthritis [6]. Chicken type II collagen also induced oral tolerance in RA patients and reduced the inflammatory responses [7].

Another important issue refers to the relevance of administration of tolerogenic citrullinated peptide at initial stages of ACPA detection prior to the clinical symptoms of arthritis for prevention of the arthritis or even to reduce disease severity.

We believe that specific peptide therapy may provide an opportunity to divert patients with an incoming inflammatory condition from developing diseases such as RA, furthermore such an approach seems to be specific avoiding unnecessary exposure to nonspecific immunomodulatory agents.
